# Evaluation of Microencapsulation Techniques for MICP Bacterial Spores Applied in Self-Healing Concrete

**DOI:** 10.1038/s41598-019-49002-6

**Published:** 2019-08-28

**Authors:** Wiboonluk Pungrasmi, Jirapa Intarasoontron, Pitcha Jongvivatsakul, Suched Likitlersuang

**Affiliations:** 1 Associate Professor, Department of Environmental Engineering, Faculty of Engineering, Chulalongkorn University, Phayathai Road, Pathumwan, Bangkok 10330 Thailand; 2 Research Network of NANOTEC-CU on Environmental, Department of Environmental Engineering, Chulalongkorn University, Bangkok, 10330 Thailand; 3 Master student, Department of Environmental Engineering, Faculty of Engineering, Chulalongkorn University, Phayathai Road, Pathumwan, Bangkok 10330 Thailand; 4 Assistant Professor, Innovative Construction Materials Research Unit, Department of Civil Engineering, Faculty of Engineering, Chulalongkorn University, Phayathai Road, Pathumwan, Bangkok 10330 Thailand; 5 Professor, Centre of Excellence in Geotechnical and Geoenvironmental Engineering, Department of Civil Engineering, Faculty of Engineering, Chulalongkorn University, Phayathai Road, Pathumwan, Bangkok 10330 Thailand

**Keywords:** Applied microbiology, Geochemistry

## Abstract

Concrete cracks must be repaired promptly in order to prevent structural damage and to prolong the structural life of the building (or other such construction). Biological self-healing concrete is a recent alternative technology involving the biochemical reaction of microbial induced calcium carbonate precipitation (MICP). This study determined the most appropriate technique to encapsulate spores of *Bacillus sphaericus* LMG 22257 with sodium alginate so as to protect the bacterial spores during the concrete mixing and hardening period. Three techniques (extrusion, spray drying and freeze drying) to encapsulate the bacterial spores with sodium alginate were evaluated. The freeze-drying process provided the highest bacterial spore survival rate (100%), while the extruded and spray-dried processes had a lower spore survival rate of 93.8% and 79.9%, respectively. To investigate the viability of microencapsulated spores after being mixed with mortar, the decomposed urea analysis was conducted. The results revealed that the freeze-dried spores also showed the highest level of urea decomposition (metabolic activity assay used as a surrogate marker of spore germination and vegetative cell viability). Thus, the self-healing performance of concrete mixed with freeze-dried spores was evaluated. The results showed that the crack healing ratio observed from the mortar specimens with freeze-dried microencapsulated spores were significantly higher than those without bacteria. This study revealed that freeze drying has a high potential as a microencapsulation technique for application to self-healing concrete technology.

## Introduction

Concrete is the most consumed and widely used construction material in the world due to its high compressive strength, durability, cost-effectiveness, design flexibility and fire resistance^1^. Nevertheless, cracks in concrete are one of the major problems that affect the strength and durability of concrete-built structures. The formation of cracks may cause the collapse of the construction building, and so repairing cracks in the concrete structure could increase the structure durability and safety as well as prolong the service life. Many traditional methods have been used for repairing cracks, like sealing with epoxy or latex binding agents, stitching, overlay and grouting. However, they are costly, decrease the aesthetic appearance, require labor work and impact the environment. In addition, the cement industry is one of the largest sources of carbon dioxide (CO_2_) emission (about 8% of global CO_2_ emission) and air pollution from the decomposition of carbonate, which is involved in the chemical reaction, and fossil fuel combustion to obtain the required high temperature (~1000 °C) to decompose the carbonate. Moreover, chemical binding materials, such as acrylic, polyvinyl acetate and butadiene styrene, have health concerns due to their toxicity^2^. Thus, an eco-friendly remediation method using a self-healing concept is highly desirable.

Self-healing concrete is the ability of concrete to repair its small cracks autonomously and has recently become of interest in civil engineering. Several processes are proposed for the concept of self-healing concrete technologies, and are grouped as (1) natural, (2) chemical and (3) biological processes^3^. However, biological self-healing concrete is regarded as an environmentally friendly and economical technology, with the potential for diverse engineering applications, such as to remove heavy metals and radionuclides^4^, CO_2_ sequestration^5^, microbial enhanced oil recovery^6^ and restoration of construction materials, such as soil, limestone and concrete^7–9^.

Microbially induced calcium carbonate (CaCO_3_) precipitation (MICP) is performed by alkalophilic microorganisms and involves ureolytic activity, also known as bio-mineralization, in the space between a crack by mixing a bacterial spore, nutrients and calcium ion solution into the concrete^10^. During MICP process, CaCO_3_ crystals are deposited onto the surface of the crack and then accumulate until the crack is filled. Owing to the alkaline environment of the concrete (pH 12–13) and the difficulty in estimating the cracking time, the use of spores of alkali-tolerant bacteria, such as *Bacillus* species, is more advantageous than using vegetative cells for incorporation into concrete because spores have a much longer shelf life and higher resistance against the harsh conditions^11,12^.

The relevant bacterial spores and other required agents (nutrients and precipitation precursor) are added into the concrete during the mixing process. When cracking of the concrete occurs the embedded spores in the cracked zone would be activated by the moisture and O_2_ exposure, leading to their enhanced metabolism with the precipitation of CaCO_3_ to heal the cracks^13^. However, direct incorporation of bacteria into the concrete, dramatically reduces the microbial metabolic activity^14^, and so microencapsulation is essential to enhance the bacterial viability in the extreme conditions for a longer period of time. In this approach, the bacterial spore is encapsulated in a protective carrier, like a polymer or microcapsule^15^ that can resist the high pH and humidity limitations of concrete, including the mechanical forces during the concrete preparation processes^16^. On the other hand, they should easily break open when cracks appear so as to release the spores to germinate and subsequently precipitate CaCO_3_ to heal the cracks^17^.

In practice, microencapsulation technologies have mostly been applied for medical treatment, pharmacy applications, food industry and agricultural fertilizer. However, this technology can likely be used for the MICP of bacterial spores in concrete to ensure their viability and metabolic activity during long term usage in concrete. Previous studies have only focused on the crack healing efficiency, while the viability of the bacteria remains unknown. The objective of this study was to determine a suitable microencapsulation technique to preserve the bacterial spores of *Bacillus sphaericus* LMG 22257 incorporated in self-healing concrete. The experimental work consisted of comparing the performance of three techniques to encapsulate the bacterial spores (extrusion, spray drying and freeze drying) in terms of the spore survival rate and encapsulation yield (EY). The viability of the bacteria after the mortar preparation process was evaluated by their urease activity. The results of this study indicate the most appropriate microencapsulation technique for self-healing concrete.

## Materials and Methods

### Bacterial spore preparation

*Bacillus sphaericus* LMG 22257 (Belgian Coordinated Collection of Microorganisms, Ghent) was used in this study and was grown in YU medium (Nutrient broth 3 g/L, NaHCO_3_ 2.12 g/L and urea 10 g/L) that had been autoclaved at 121 °C for 15 min before use. The cultures were incubated at 30 °C for 7 d with agitation at 100 rpm. The vegetative bacterial cells were then induced to form spores by heat shock treatment at 80 °C for 10 min in a water bath and were then immediately cooled in a crushed ice water for 5 min. Germination of the spores was induced by culturing in YU medium at 30 °C with agitation at 100 rpm for 2 d to confirm that more than 90% of the cells were spores. The spores were then harvested at 4 °C by centrifugation 8,000 rpm (2,862 × g with 4 cm. rotor radius) for 15 min. The spore suspension was washed twice with 0.1% (w/v) NaCl-peptone buffer and then stored in sterile distilled water at 4 °C. Noted that after harvesting, the spore suspension was washed with NaCl-peptone buffer to provide an isotonic environment. This is of particular importance when trying to recover cells or spores that may be stressed or sensitive to osmotic pressure.

To ensure that most of cells become spores after the heat-shock step and spore germination process, microscopic examination and endospore gram staining were evaluated. The number of spores were compared before and after these inducing steps. Malachite green staining, a specific stain of endospores, clearly confirmed that sporulation occurred. The proportion of red rod-shaped bacterial cells that contained a green space (spore) within the terminal region of the cell indicated that an average of 90% of the cells were present as spores.

### Microencapsulation of the bacterial spores

In each microencapsulation technique, 2% (w/v) spore suspension, which contained 10^6^ cells/mL in 200 mL of sodium alginate solution was used. The details of each technique are provided as follows:

#### Extrusion technique

The spores were suspended to a homogeneous suspension in 200 mL of 2% (w/v) sodium alginate solution and conveyed in a silicone tube, which compresses the force of the peristaltic pump at 120 rpm, to a syringe needle (6 mm diameters) for extrusion as free-fall droplets into 2% (w/v) CaCl_2_ solution and left to harden at room temperature for 30 min. The solid capsules formed were washed with sterile distilled water, dried on clean paper until completely dry and then stored in a desiccator.

#### Spray drying technique

A pilot-scale spray dryer (BUCHI™ B-290, Switzerland) was used in this study. The inlet-air was heated to 105 °C after passing through a blower. A peristaltic pump delivered the mixed spore and 2% (w/v) sodium alginate solution to a fluid stainless-steel atomizer. The outlet-air temperature (73 °C) was controlled by adjusting the flow rate of the feed solution and aspiration rate of 10% and 100%, respectively. Dried capsules were collected from the vessel of the cyclone then kept in a tightly sealed bottle and stored in a desiccator.

#### Freeze drying technique

Before sublimation, the spore suspension in 2% (w/v) sodium alginate solution was frozen at −46 °C in liquid ethanol and then the mixed solution was freeze dried for 24 h in a laboratory-scale freeze dryer (Christ Alpha 2–4/LD Plus, Germany) at a condenser temperature below 0 °C and a chamber pressure of 0.05 mbar (5 Pa). The dried products in sheet form were spun to small pieces and stored in a desiccator.

### Determination of the particle morphology by scanning electron microscopy (SEM)

The microcapsules were analyzed using a scanning electron microscope in order to examine the external appearance of the particles. All specimens were prepared by mounting to the carbon conductive adhesive tape on SEM stub holder then sputtered with gold coating in a Hummer IV sputter coater. SEM photographs were taken by the scanning electron microscope (JEOL, JSM-IT300 InTouchScope^TM^, USA) at a magnification 100x to 10,000x, at room temperature, equipped with an X-ray detector model with an operating of 10 kV.

### Survival of the bacterial spores after the microencapsulation process

Microencapsulated bacterial spores were enumerated by the standard plate count method as colony forming units (CFU)/mL derived from triplicate platings at each dilution. The encapsulation yield (EY), which is a measurement of the efficacy of entrapment and survival of viable spores during the encapsulation process, was calculated from Eq. (1);

1 $${\rm{EY}}( \% )=\frac{N}{{N}_{0}}\times 100 \% $$

where *N* is the number of viable entrapped spores and *N*_0_ is the number of initial spores before the encapsulation process.

### Mortar specimen preparation

**Figure 1:**
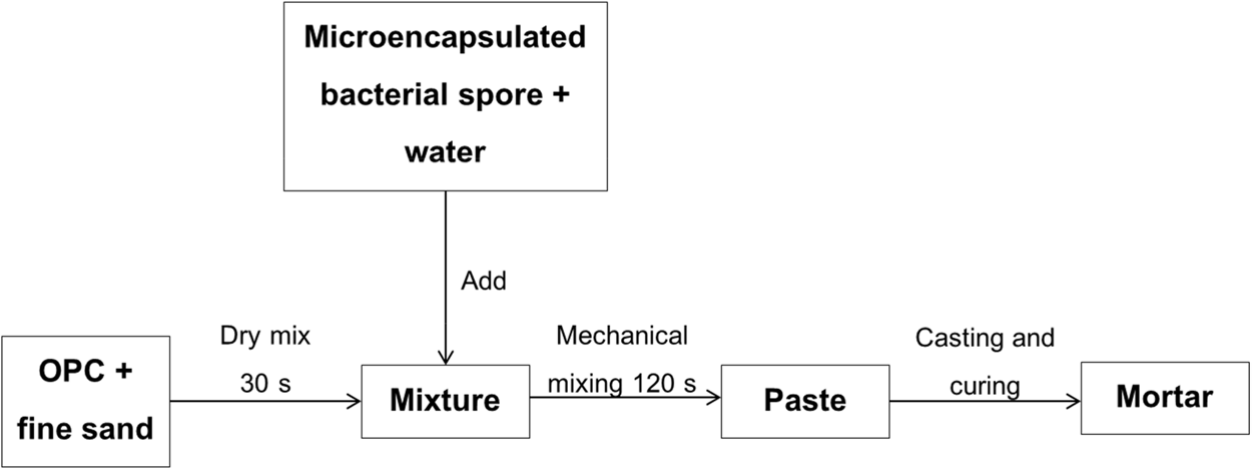
Preparation of the mortar with microencapsulated bacterial spores. OPC = ordinary Portland cement type 1.

Mortar specimens (50 mm × 50 mm × 50 mm), with different types of sodium alginate encapsulated bacterial spores, were cast to investigate the viability of the microencapsulated bacterial spores after being mixed with mortar. The specimens were made with a water per cement weight ratio of 0.5 and a sand per cement weight ratio of 1:3 using ordinary Portland cement type I, fine-grained sand and tap water. The number of microcapsules added was based on the capsule dry weight as 2% (w/w of cement). The microcapsules were mixed into the mortar together with water. The mixing procedure for mortar is in accordance with ASTM C305-14^18^. The mixing period was about 150 s, which is appropriate to merge all the ingredients into a homogeneous state. After casting, all molds were put in an air-conditioned room with the controlled temperature of 23.0 ± 2.0 °C and the relative humidity of 60–70% and then de-molded after 24 h. Although the samples were not cured in the moist room for the first 24 h, they were immediately submerged in water after de-molding for another 24 hours to maximize the hydration. After curing, the mortar specimens were pounded into pieces. The specimen preparation is illustrated in Fig. 1.

Noted that the spray dried and freeze dried microcapsules were first hardened with CaCl_2_ solution for 30 min before mixed into the mortar. The favorable qualifications for encapsulation of bacterial spores are water insoluble and hard, which can protect or preserve the spores for a long time. Moreover, the capsules should not dissolve in the water that is used for making the cement mortar. Since sodium alginate is water soluble, the most common method to prepare capsules from an aqueous alginate solution is to combine it with ionic cross-linking agents, such as divalent cations (i.e., Ca^2+^). For the extrusion, the spore suspension in sodium alginate was extruded through a syringe needle in the form of droplets into the CaCl_2_ hardening solution, whereupon the droplets become insoluble capsules that are hard and non-water dissolved. In contrast, encapsulated spores prepared by spray drying and freeze drying techniques do not have any cross-linkages with CaCl_2_, hence these capsules from spray dried and freeze dried method had to harden with a CaCl_2_ solution before mixed into the mortar.

### Viability of the bacterial spores in mortar

The viability of the bacterial spores in mortar was determined indirectly from the urease activity they secreted, based on the principle that urea forms a yellow-colored complex with *p*-dimethylaminobenzaldehyde in a low acidic solution at room temperature. The amount of hydrolyzed urea that occurred by bacterial activity was thus determined by measuring the light absorbance at 422 nm by spectrophotometry.

**Figure 2:**
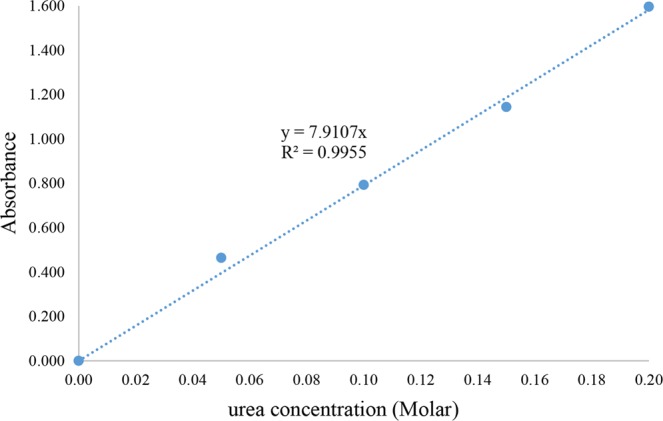
The linear calibration curve of urea standard solution.

In this study, the mortar specimens were pounded into pieces and then selected for fine particles by screening using sieve no. 20 for extruded and freeze dried specimens, while sieve no. 400 was used for spray dried specimen. The amount of hydrolyzed urea was determined spectrophotometrically as reported^19^. In brief, 10 g of the fine mortar was added to 100 mL YU medium that contained 0.170 M urea and was incubated for 8 h. A mixture (0.5 mL) containing 4% (w/v) of *p*-dimetylaminobenzaldehyde and 4% (v/v) sulfuric acid in pure ethanol was then added to 2 mL of the mortar suspension and incubated at room temperature (25 °C) for 10 min, whereupon the absorbance was measured at 422 nm (A_422_) against that of a standard urea solution, a set of solutions of YU-medium that contained different known concentrations of urea ranging from 0 to 0.2 M. These different urea concentrations were reacted with *p*-dimethylaminobenzaldehyde in a low acidic solution to form a yellow-colored complex at room temperature. The light absorbance of each solution was then measured at 422 nm in order to prepare a linear calibration curve as shown in Fig. 2. The remaining urea concentration in the sample was evaluated using the linear calibration curve. The hydrolyzed urea was then calculated from Eq. (2),

2 $${\rm{Hydrolyzed}}\,{\rm{urea}}({\rm{M}})={\rm{initial}}\,{\rm{urea}}({\rm{M}})-{\rm{remaining}}\,{\rm{urea}}\,({\rm{M}})$$

Noted that the initial urea concentration in YU medium was 10 g/L or 17 µM, while the remaining urea was derived from the linear calibration curve.

### Statistical analysis

Numerical results are shown as the mean ± standard error (SE). Significance of any differences in the means of the EY and amount of hydrolyzed urea was evaluated by analysis of variance (ANOVA) or Kruskal-Wallis test (KWT), accepting significance at the p < 0.05 level. All statistical analysis was performed using the SPSS statistics software.

### Ethical approval

This article does not contain any studies with human participants or animals performed by any of the authors.

## Results

### Morphology of the capsules

**Figure 3:**
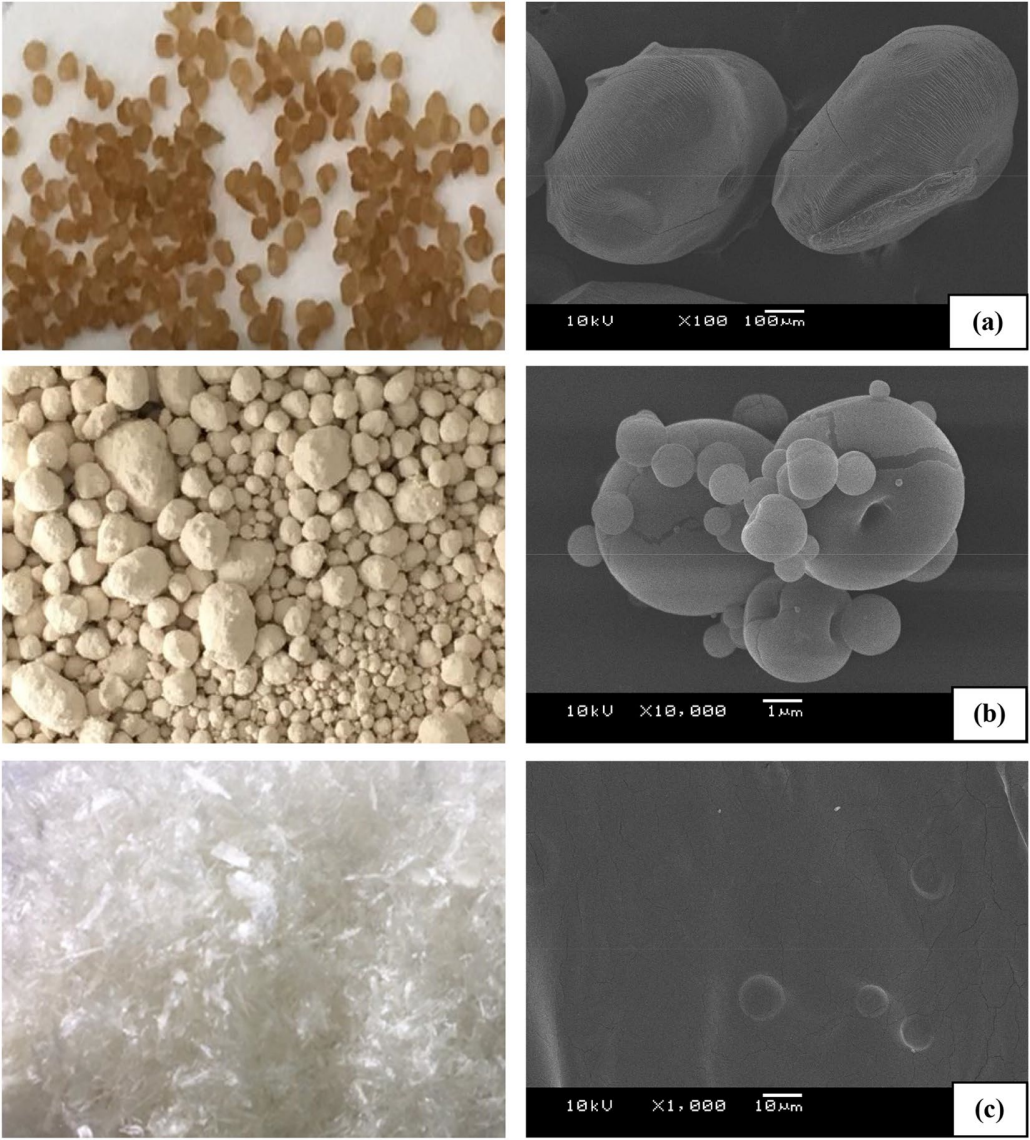
Representative physical and SEM images of the encapsulated capsules; showing the: (**a**) extruded capsule at 100x magnification, (**b**) spray-dried capsule at 10,000x magnification and (**c**) freeze dried capsule at 1,000x magnification. Images shown are representative of those seen from at least 3 such fields of view per sample and 3 samples.

Figure 3 showed the different shapes and morphologies of the capsules after the three different microencapsulation techniques. The extruded capsules (Fig. 3a) were nearly spherical in shape, but the surface was rough and had several wrinkles. Their size ranged from 600–800 µm diameter. The spray dried capsules (Fig. 3b) were similar to the powder and varied in size between 1–10 µm diameter, and most of these capsules were of a smooth spherical shape with a dimple. On the other hand, the freeze dried capsules (Fig. 3c) were irregular in shape and morphology and so a definite size could not be specified. The shape, however, was a sheet form that looked like a scaffold foam with multi-cavities distributed throughout them. The SEM image of the freeze dried capsules in Fig. 3c showed that the spores bulged within the sodium alginate coat, meaning that these spores were probably entrapped successfully. Moreover, indirect investigation by a spore viability test confirmed and ensured that the spores were protected and successfully encapsulated. As all particles were bulged, no particles were observed to be comprised of coating materials only.

### Encapsulation yield of the microencapsulated bacterial spores

In this study, the encapsulation yield (EY) of the encapsulated spores was evaluated by the standard plate count method, which assumes each colony arises from a single cell (or spore), recording it as colony forming units (CFU)/mL. The results of standard plate count and encapsulation yield (EY) measurements are summarised in Table 1, which represent the effect of the microencapsulation process on the bacterial spores and degree of preservation of their viability. The amount of viable bacterial spores after encapsulation is somehow bigger than that before encapsulation, since the bacterial cells/spores after encapsulation were more evenly distributed (less clumped than before encapsulation) in the suspension to be plated. The data showed that the encapsulation process reduced the viability of the bacterial spores. Freeze dried spores give the highest EY (100%), whilst the EYs of the extruded and spray-dried spores were 93.8% and 79.9%, respectively. In comparison, the EYs of each microencapsulation technique were not significantly different (p < 0.05; n = 3).

**Table 1:** Viable bacterial spore and encapsulation yield.

Microencapsulation techniques	Viable bacterial spores × 10^6^ (CFU/mL)		Encapsulation yield (EY) (%)
	Before encapsulation	After encapsulation	
Extrusion	2.26 ± 0.87	2.12 ± 0.32	93.8
Spray drying	2.34 ± 0.31	1.87 ± 1.51	79.9
Freeze drying	1.37 ± 0.48	1.39 ± 0.67	100.0

Data are shown as the mean ± SE, derived from three replications. No means were significantly different (p > 0.05; KWT).

### Viability of the microencapsulated bacterial spores in mortar

To investigate the viability of microencapsulated bacterial spores after mixing with mortar, the level of urea hydrolysis (urease activity) was measured. This indirect spectrophotometric measurement at A_422_ was used to reveal the number of viable (germinated) spores based upon the direct release of urease by viable bacteria that then catalyzes the hydrolysis of urea. Thus, the hydrolyzed urea refers to the bacterial spore ureolytic activity. If the amount of hydrolyzed urea is high, it indicated more metabolically viable cells and hence that the microencapsulated bacterial spores can survive and remain viable after being mixed in the mortar specimens. The hydrolyzed urea increases depending on time. During the first phase (lag phase), the bacterial spores adjust to the environment, undergoing metabolic changes and storing nutrients prior to subsequently germinating as a vegetative cell or cell active form. The ureolytic activity during this initial phase is quite low. When the bacterial cells enter the exponential or log phase, when the cells were dividing, the cells are metabolically active and synthesize and secrete urease, thereby the ureolytic activity was high then the hydrolyzed urea increased.

**Figure 4:**
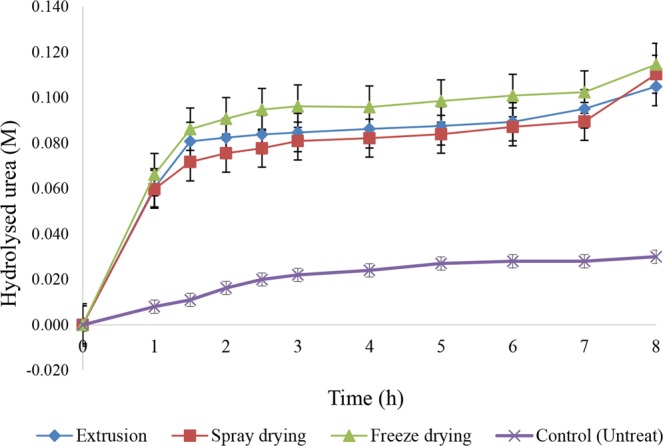
Hydrolyzed urea concentration (M) by microencapsulated bacterial spores in mortar. Data are shown as the mean ± SE, derived from three replications. For the three encapsulation methods, no means were significantly different to the others (p > 0.05).

In general, the encapsulation appeared to protect the bacterial spores during the mortar mixing and hardening period as shown in Fig. 4. The results showed that the freeze dried spores could decompose urea at 0.115 M (67.4%), while spray dried and extruded spores decomposed urea at 0.110 M (64.9%) and 0.105 M (61.7%), respectively. Thus, some two-thirds of the bacterial spores in all three microencapsulation techniques remained viable and able to germinate into active vegetative cells. That is the ureolytic activity implied that some 61–67% of the encapsulated spores were not damaged during the mixing and casting of the mortar as well as that they can tolerate the unsuitable conditions of the mortar. The urea decomposition value (urease activity) of spores from each microencapsulation technique were not significantly different (p < 0.05; n = 3).

### Application of the freeze-dried microencapsulated bacterial spores on concrete healing

The biological self-healing process of concrete is mainly mediated by the microbial precipitation of CaCO_3_, and so measurement of the repaired part of the crack can indicate the self-healing efficiency. The main purpose of this work is to evaluate the performance of microencapsulated bacterial spores on the self-healing of concrete by crack filling, which was assessed by the visualization of the crack area before and after healing. The 50-mm^3^ mortar samples were prepared as in the viability test. The samples were prepared with the addition of 2% (w/w of the cement weight) of freeze dried capsules, nutrient and calcium ion source. After 28 d curing, cracks were induced in the mortar samples by subjecting them to a monotonic compression test. All mortar samples were incubated in a wet-dry cycle in water for 7 d. In this research, a wet-dry cycle incubation was used to simulate the moisture changes in concrete structure due to rain or water. During the wet period, the specimens could absorb enough water, which can remain some moisture in the intercellular matrix during the dry period. When the specimens were exposed in the air, more oxygen becomes available for the bacteria. A wet-dry cycle can accelerate the diffusion of the nutrients from the intercellular matrix to the surficial crack zone without excessive leaking to the bulk solution. This process can also keep the specimens with enough water for bacterial activities during the dry period.

**Figure 5:**
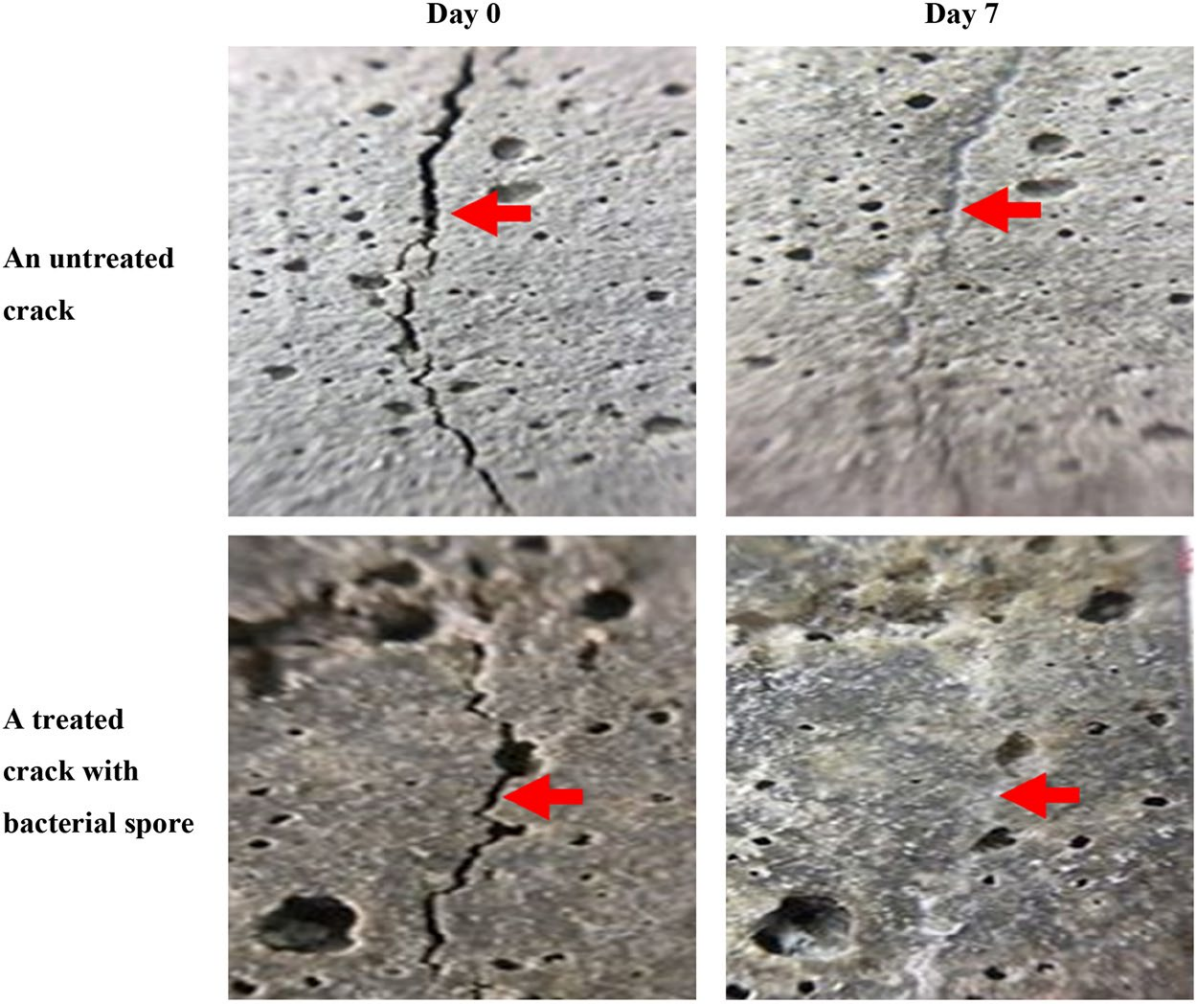
Crack-healing activity in mortar by sodium alginate microencapsulated bacterial spores formed by freeze drying.

The progress of the crack healing process was followed by the images of the crack area taken at each indicated time interval. The healing ratio of the initial and final crack area in the images were determined by the image processing. Examples of crack areas are shown in Fig. 5, where it was obviously indicated that the crack can be gradually healed with the increase of time. The crack width measurement are also summarised in Table 2. The crack area of the mortar specimen without microencapsulated bacterial spores gave a healing ratio of 72.7%, which was less than that with the encapsulated bacterial spores (95.4%). Thus, bacterial spores encapsulated by sodium alginate via freeze drying can enhance the crack healing efficiency by their biochemical activity and so can possibly be used as a microencapsulation technique for MICP spores for application in concrete technology.

**Table 2:** Crack width measurement.

Mortar specimen	Crack width (mm)	
	Day 0	Day 7
Untreated	0.19 ± 0.04	0.12 ± 0.09
Treated with bacterial spores	0.28 ± 0.03	0.02 ± 0.02

Data are shown as the mean ± SE, derived from three replications. Three measurements were made for each replication with a spacing of 1 mm. No means were significantly different (p > 0.05; KWT).

## Discussion

The viability of bacteria is the most crucial issue in applying MICP for self-healing concrete technology. Microencapsulation of bacterial spores with a protective carrier offers a solution to the problem of an otherwise low cell viability in a concrete environment. In this study, the potential microencapsulation techniques were comparatively studied. The three techniques to prepare sodium alginate encapsulated bacterial spores (extrusion, spray drying and freeze drying) had the potential to protect and preserve bacteria in the concrete mixing and hardening environments. Freeze drying provided the highest spore survival rate and viability, which is because the freeze-dried capsules were less influenced by the encapsulation process and had a low rupture rate during the concrete mixing and hardening stage. In addition, freeze-dried capsules still performed their biological activity to induce CaCO_3_ precipitation for the self-healing of concrete. Therefore, freeze drying can possibly be used as a microencapsulation process for MICP spore applications in concrete technology. The key discussions from the study can be pointed out as follows.

### Effect of the microencapsulation method on the morphology and survival rate of the bacterial spores

The morphology and survival of the microencapsulated bacterial spores depends on the condition of the encapsulation process. The capsule characteristics and stability of the bacterial spores may be affected by the temperature, moisture, water content and microencapsulation shell material. For the extrusion technique, the shape and size of the extruded gel bead depends on the nozzle diameter and the distance between the nozzle and the CaCl_2_ solution. A very limited type of shell material is available for the extrusion technique^20^. However, the extrusion procedure is still simple, cost-effective and cannot damage the bacterial spores and so results in a high cell survival level because the technique does not use harmful solvents, and perfectly encapsulates the spores under room temperature conditions^21^. However, after these extruded gel beads were dried, the loss of water from the shell material (sodium alginate) made the capsule surface become rough and wrinkled. In addition, the capsule size was rather large (typically 500–1000 µm), which can become a hindrance in concrete application. Moreover, the extrusion process is not appropriate for industrial scale production due to the slow microsphere formation and low production capacity.

For the spray dried capsules, a viscous film appeared on the external surface of the droplets caused by the rapid evaporation of the solvent during the early stage of the spray drying. This film could impede the diffusion of water to the periphery of the droplet. When the chamber temperature rose up to 105 °C, the water vapor pressure inside the droplet increased rapidly. When at a critical temperature (73 °C), the film would burst and then collapse, resulting in capsules with dimples or holes. A higher outlet air temperature in a spray dryer promotes a faster drying rate and influences the capsule morphology, such that capsules collected with a higher outlet temperature had dimples and holes, while capsules collected with a lower outlet temperature were more spherical without any dimples^22^.

In spray drying technique, there are several advantages, including the uncomplicated control of microcapsule characteristics by changing the operating parameters, its economic efficiency, and convenience to scale-up^23^. However, some spray drying operating conditions, for instance the inlet and outlet air temperature, feed flow rate and suction power, impact negatively on the bacterial cell survival. The optimum inlet and outlet air temperatures affect the obtained EY, where a higher inlet temperature can damage or dehydrate the heat sensitive components in capsules^24^. During the spray drying process, heat and dehydration stresses have significant adverse effects on the bacterial cell survival. Both stresses cause cellular damage and then permanent loss of cell viability^25,26^.

Dehydration stress mainly induces shrinkage of the cell membrane, due to the rapid removal of water that initiates changes in its fluidity and causes the loss of cytoplasmic volume and leakage of intracellular components^27^. Whereas, heat stress acts more on the denaturation of important cellular structures, for instance the ribosomes, proteins, enzymes and genetic material. Previous work on the spray drying of *Lactobacillus bulgaricus* showed that ribosome melting occurred at about 60 °C, while heating at 65 °C resulted in damage to protein of the cytoplasmic membrane and enzymes, and at 90 °C the genetic material (DNA and RNA) was denatured^28^. If the inlet air temperature and suction power are low, the yield of microcapsules will be reduced. This may be because the water emulsion cannot completely evaporate, which results the formation of high moisture content microcapsules, poor liquidity and ease of agglomeration. Moreover, with aforementioned conditions, an additional large amount of capsules still remain and attach on the surface of the drying chamber or the cyclone, and so they could not be collected^29^.

For the freeze-drying technique, the factors that predominantly influence the structure of the obtained capsule are the type and amount of coating material. In this research, the naturally gelatin agent, as sodium alginate, was used. The achieved freeze dried capsules were similar to a foam sheet with multi-cavities distributed throughout its structure. In accord, this was also observed in *Bifidobacterium* BB-12 encapsulated capsules^30^. The uniformly porous cavities were reported to be the residual hollows formed from the vaporized ice crystals that were distributed inside the capsules and then sublimed^31^. The freeze-drying technique is commonly employed for the storage and preservation of microorganisms in both laboratory and industrial applications.

The optimal performance of the preservation process should optimize the survival potential of the bacteria and their spores and stabilise their metabolic activity. The bacterial cells are subjected to osmotic stress through the reduction in the water activity (A_w_) of the medium as well as the external accumulation of solutes. However, bacterial cells lose their water slowly by sublimation and so the cell membrane is not damaged by osmotic shock^32^. In addition, the sublimation, which usually occurs during the freeze drying process, causes less oxidative shock damage since there is no metabolic stress due to the low rates of chemical reaction and the low oxygen concentration during sublimation from a cold environment^33^. Anyway, the moisture content of the freeze dried capsules is an essential factor that affects the stability of the bacteria during storage. In general, microorganisms can survive better at low A_w_ values of about 0.2^34,35^. This amount of water is sufficient to stabilise the microcapsules, leaving sufficient free water available for biochemical reactions and prolonging their shelf life^29^. However, freeze dried bacteria still retained 3–4% (w/w cell weight) of water, which was sufficient to promote the required enzymatic reactions for the cells to revive again^36^.

### Viability of the microencapsulated bacterial spores in mortar

Once added in the concrete paste, the encapsulated bacterial spores must endure the following improper conditions: (1) lack of germination inducers, such as nutrients, water and oxygen, within the mortar, (2) high mechanical shear force and compressive power during the concrete mixing and curing, respectively, and (3) the extreme alkaline pH and high temperature in the mortar. These factors may cause the encapsulated bacterial spores to lose their viability. Bacterial spores shall be protected to tolerate the concrete mixing and casting procedures as well as the long-term periods in an inactivated state embedded in the concrete prior to cracking. Our results showed that MICP encapsulated bacterial spores were still viable. Therefore, microencapsulation is a potential alternative way to preserve bacterial spores for concrete self-healing. However, a proper approach for this application is required in order to protect the active MICP spores under the severe conditions used for the concrete mixing and hardening processes. The encapsulated cells must not be ruptured and release the bacterial spores before cracks occur.

Moreover, the coating material should not be too strong^37^. For these reasons, we focused on alginate, a kind of hydrophilic gel with networking of polymer chains and thermo-irreversible. Alginate hydrogel has a high capacity of water absorption and can prolong a significant amount of water inside its network without dissolving. This absorbed water would then be slowly discharged to the surrounding zone. Thus, these traits of alginate make it suitable as a carrier agent for protecting the bacterial spores from the cement mixing, casting and hydration processes. Moreover, its swollen feature can sustain an internal water storage for spore germination and initiate the bacterial activity as well as help the CaCO_3_ precipitation when cracking in the concrete occurs.

The alginate encapsulated MICP spores obtained by extrusion or spray drying were quite rigid and rather strong, like sand beads. Consequently, they were difficult to break open and release the spores for germination. When cracks formed in the concrete, these bacterial spores do not revive and no reactions occurred. Whereas, the freeze-dried capsules were weaker and more easily broken when the concrete cracked. Thus, the bacterial spores receive the germination inducers and can revive again.

Although the encapsulation techniques enhanced the survival of bacterial spores, the shear stresses from the concrete mixing may eventually destroy the capsules and reduce the spore viability. Since the shear stress refers to the ratio of the concrete mixer blade shear force and the surface area of the microcapsule, the round-shaped encapsulated capsules obtained from the extrusion and spray drying methods will be affected to a greater extent by the shear stress compared to the flat-shaped freeze dried capsules. Furthermore, the loss of spore viability was related to the pore size (diameter) of the concrete. After curing, cement particles become denser and the porosity becomes less than 1% of the concrete mass. The pore sizes within young concrete (3–7 d cured) were only 0.1–0.5 µm, while the bacterial cells were about 1–2 µm. Therefore, these bacterial cells were mainly compacted on the surface of the cement particles^14^. The extruded and spray-dried capsules had a larger size than the pore cavity and so they could not be inserted within these spaces and were compressed. In contrast, the flat sheet of the freeze-dried capsules were smaller and flexible and could be situated within the pores in the concrete or attached on the surface of the cement particles, and so this kind of capsule was less affected by the compressive force during the concrete hardening than the others.

Lastly, the homeostasis of pH and temperature during cement hydration can markedly reduce the bacterial cell viability. The typical conditions in cement are a pH of 12–13, temperature around 60 °C and low A_w_ , which are a particularly harsh environment for bacteria. The optimum condition for the growth of *Bacillus sphaericus* LMG 22257 cells and germination of their spores is a weak base at pH 7–9 and mesophilic temperature of 30–45 °C^38^. Thus, it was determined that the optimum pH and temperature are critical for Ureolytic activity, and the viability of bacteria decreased with increasing pH and temperature. A highly alkaline state and temperature increase will slow down the enzyme activity for growth and germination^39^. The encapsulated capsule from the freeze-dried technique still retained water at 3–4% of the cell weight, which was efficient at controlling the homeostasis and supporting cellular metabolism.
